# Novel Chemokine-Based Immunotoxins for Potent and Selective Targeting of Cytomegalovirus Infected Cells

**DOI:** 10.1155/2017/4069260

**Published:** 2017-01-30

**Authors:** Katja Spiess, Mads G. Jeppesen, Mikkel Malmgaard-Clausen, Karen Krzywkowski, Thomas N. Kledal, Mette M. Rosenkilde

**Affiliations:** ^1^INAGEN Aps., Kongens Lyngby, Denmark; ^2^Laboratory for Molecular Pharmacology, Department of Neuroscience and Pharmacology, Faculty of Health and Medical Science, University of Copenhagen, Copenhagen, Denmark; ^3^Section for Life Science and Bioengineering Innovation, Veterinary Institute, The Danish Technical University, Kongens Lyngby, Denmark

## Abstract

Immunotoxins as antiviral therapeutics are largely unexplored but have promising prospective due to their high selectivity potential and their unparalleled efficiency. One recent example targeted the virus-encoded G protein-coupled receptor US28 as a strategy for specific and efficient treatment of human cytomegalovirus (HCMV) infections. US28 is expressed on virus-infected cells and scavenge chemokines by rapid internalization. The chemokine-based fusion-toxin protein (FTP) consisted of a variant (F49A) of CX_3_CL1 specifically targeting US28 linked to the catalytic domain of* Pseudomonas exotoxin* A (PE). Here, we systematically seek to improve F49A-FTP by modifications in its three structural domains; we generated variants with (1) altered chemokine sequence (K14A, F49L, and F49E), (2) shortened and elongated linker region, and (3) modified toxin domain. Only F49L-FTP displayed higher selectivity in its binding to US28 versus CX_3_CR1, the endogenous receptor for CX_3_CL1, but this was not matched by a more selective killing of US28-expressing cells. A longer linker and different toxin variants decreased US28 affinity and selective killing. Thereby, F49A-FTP represents the best candidate for HCMV treatment. Many viruses encode internalizing receptors suggesting that not only HCMV but also, for instance, Epstein-Barr virus and Kaposi's sarcoma-associated herpesvirus may be targeted by FTPs.

## 1. Introduction

Immunotoxins constitute a class of protein-based therapeutics and are considered promising anticancer therapies in the clinic [1, 2]. They are chimeric molecules that consist of a toxin fused to a targeting moiety. The targeting domain is most commonly the antigen-binding fragment of a monoclonal antibody but can also comprise receptor ligands, such as a growth factor or a cytokine that targets specific cell surface receptors [1].* Pseudomonas* exotoxin A (PE) is a highly toxic protein that has been used to generate several immunotoxins undergoing evaluation in clinical trials [3–5]. The structure and mechanism of action of PE allow for modifications so that PE can be converted into an immunotoxin by changing its target to a different cell surface receptor [6]. PE-based immunotoxins usually contain a fragment of the* Pseudomonas aeruginosa* exotoxin A, consisting of domains II and III of the native toxin, while domain I is replaced by the targeting moiety. Once the target domain binds to its receptor, the immunotoxin is internalized by endocytosis, cleaved in domain II by the proprotein convertase furin, leading to the release of the toxin and a subsequent cell killing.

Within the last decades, the potential and success rate of developing anticancer immunotoxins have been translated to other indications, such as infectious diseases [1]. Recently, the first antiviral immunotoxin entered the stage, targeting the viral G protein-coupled receptor (GPCR) US28 encoded by the human cytomegalovirus (HCMV) [7]. The targeting moiety was not an antibody, but a variant of the chemokine CX_3_CL1 optimized for specific binding to US28. Wild- type CX_3_CL1 only targets one additional receptor, namely, its cognate receptor, CX_3_CR1. CX_3_CL1 is unique among endogenous chemokine ligands, as it exists in two forms: a soluble form and a form where the chemotactic chemokine is anchored to the cell membrane by an extended mucin-like stalk and an alpha-helix through the membrane [8].

Viral piracy of the endogenous chemokine system is a commonly used viral strategy to circumvent and/or manipulate the host chemokine system and thereby the host immune response in benefit of virus survival and spreading [9–11]. Thus, HCMV devotes a significant part of its genome to immune modulatory gene homologs, including several predicted 7-transmembrane GPCRs: UL33, UL78, US27, and US28, with US28 being a functional chemokine G protein-coupled receptor [12]. Still related to the immune system but outside the chemokine system, herpesviruses have developed another strategy to manipulate the host by downregulation of surface expressed MHC class I molecules, a property described for the constitutively active GPCR denoted BILF1 by Epstein-Barr virus (EBV) [13–18]. However, most of the viral GPCRs show homology to the humane chemokine receptors, for example, the resemblance of US28 to the human CX_3_CR1 receptor [9, 12] and the CXC-chemokine receptors encoded by rhadinoviruses like ORF74 encoded by human Kaposi's sarcoma-associated herpesvirus (KSHV) and of* Equine herpesvirus* 2 and ECRF3 encoded by* Herpesvirus saimiri* [19–22]. In addition, viruses encode chemokine ligands, for example, vCCL1-3 encoded by KSHV [23] and MC148 from the pox virus molluscum contagiosum [24–26]. A third principle to target the chemokine system by virus is by scavenging host chemokines by viral chemokine binding proteins [27].

**Figure 1:**
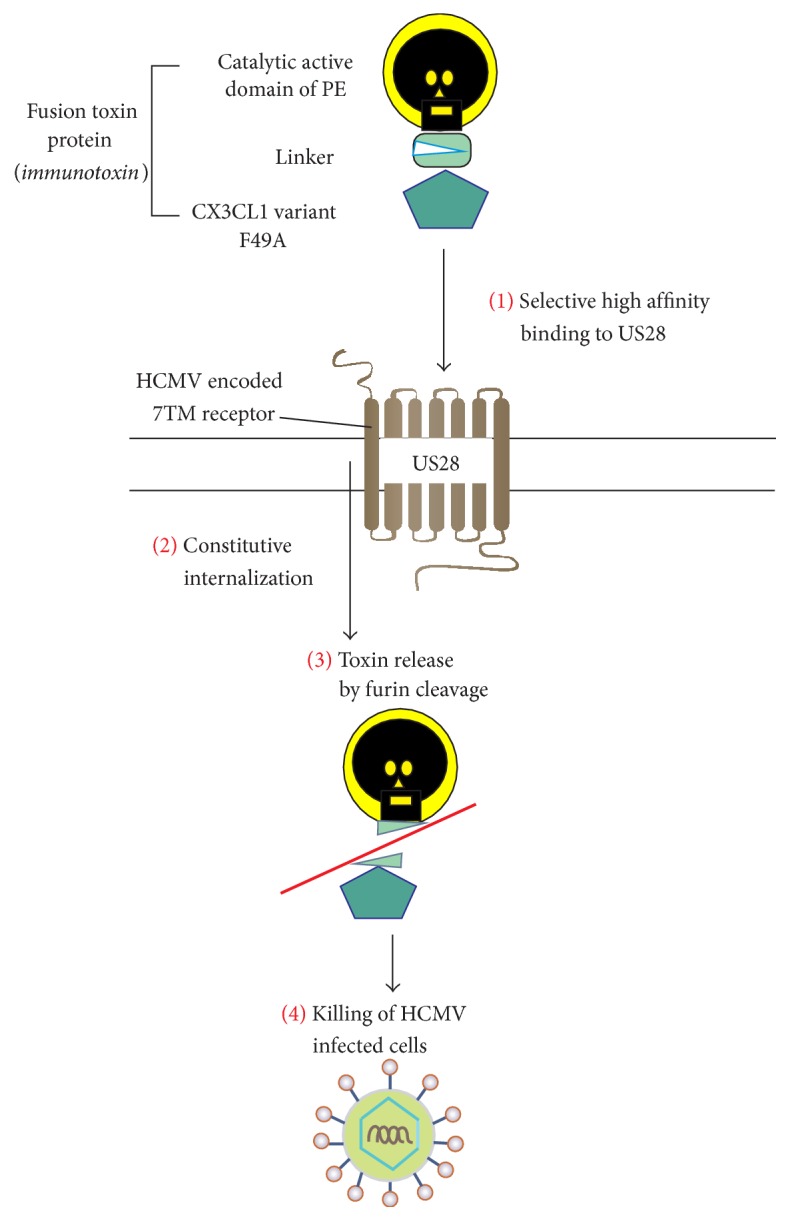
Selective killing of HCMV infected cells by F49A-FTP. The FTP consisting of the CX_3_CL1 variant F49A and the catalytic active domains of PE binds selectively to US28 (1), and the internalization of the FTP is triggered by internalization of F49A binding to US28 (2). The release of F49A is achieved by furin cleavage (3), and the protein synthesis is inhibited by PE, leading to (4) killing of the human cytomegalovirus (HCMV) infected cells.

US28 is a broad-spectrum chemokine receptor yet binds CX_3_CL1 with superior affinity as compared to CC-chemokines [28]. Moreover, it signals with high constitutive activity [29, 30] and undergoes constitutive ligand-independent receptor internalization [31], a feature well suited for immunotoxin delivery. Based on the molecular and pharmacological properties of US28 and the structural property of CX_3_CL1, the prototype immunotoxin CX_3_CL1-FTP was designed [7]. The chemokine domain of CX_3_CL1 was chosen as targeting moiety and the mucin-like stalk of CX_3_CL1 was replaced by catalytic active domains of PE. Moreover, as CX_3_CL1 also binds to the human CX_3_CR1, a US28 selective FTP was generated (F49A-FTP) (Figure 1), by introducing a single point mutation (Phe^49^ to Ala) in CX_3_CL1 [7]. Both FTPs were highly efficient in controlling HCMV infections in vitro and F49A-FTP provided unparalleled potency compared to the gold standard ganciclovir in vivo [7].

These promising results suggest that chemokine-based FTPs can be developed into therapeutics to treat HCMV-associated diseases. Here, we investigate if the US28 selective FTP (F49A-FTP) can be further optimized in terms of increased selectivity or potency by a systematic approach modifying the US28-targeting part (i.e., the chemokine), the catalytic active domains of PE, and alternations in the linker region.

## 2. Materials and Methods

### 2.1. Antiviral Fusion-Toxin Proteins (FTPs)

The FTPs were prepared as described previously [7]. Briefly, the FTP DNA constructs were cloned into the pET21a(+) vector (Novagen) and transformed into* E. coli* BL21(DE3) pLysS cells (Novagen) for the preparation of inclusion bodies. Protein expression was induced with 0.5 mM isopropyl *β*-D-1-thiogalactopyranoside (IPTG). Cells were ruptured by sonication in the following buffer: 50 mM Tris-HCl pH 8.0, 100 mM NaCl, 5 mM EDTA, 10 mM MgCl_2_, 1 mM benzamidine, 3 mM DTT, 1 mM PMSF, and 10 *μ*g/mL DNase I. The suspension was centrifuged and the pellet was resuspended and washed twice with 50 mM Tris-Cl pH 8.0, 300 mM NaCl, 0.25% sodium deoxycholate, and 5 mM DTT (the second time without sodium deoxycholate).

#### 2.1.1. Downstream Purification of Recombinant FTPs

100 *µ*L denaturation buffer (3 M GnHCl, 100 mM Tris-Cl pH 8.0, 5 mM EDTA, and 5 mM DTT) was added to solubilize the inclusion bodies, followed by incubation and centrifugation. The inclusion bodies were dialyzed against 1x PBS at 4°C and then overnight against 1x PBS containing 0.2 mM cystine and 1 mM cysteine. The protein sample was added to two sample volumes of 50 mM Tris-Cl pH 8.0 while stirring, followed by addition of 3 sample volumes of 50 mM HEPES pH 7.2, 1 mM CaCl_2_, and 5 mM MgCl_2_ and confirmation of the mixture reaching pH 7.2. The sample was filtered and loaded onto a Source 30Q column equilibrated in buffer A: 50 mM HEPES pH 7.2, 1 mM CaCl_2_, 5 mM MgCl_2_, and 50 mM NaCl. Bound protein was eluted with a gradient from 0 to 40% buffer B; buffer B is the same as buffer A, but with addition of NaCl to 1 M, and the fractions with the protein of interest were concentrated. The sample was centrifuged and loaded onto a Superdex 75PG column, equilibrated in 1x PBS.

### 2.2. Tissue and Virus Culture

Human lung fibroblasts cells MRC-5 (ATCC CCL-171) were purchased from the American Type Culture Collection (ATCC). The stable inducible clones of US28 and CX_3_CR1 were kindly provided by Hjortø et al. (Department of Neuroscience and Pharmacology, University of Copenhagen) [32]. MRC-5 cells were grown at 10% CO-2 and 37°C in Dulbecco's modified Eagle's medium (DMEM) supplemented with 10% fetal bovine serum (FBS) and 180 units/mL penicillin. The stable clones of inducible US28 and CX_3_CR1 HEK-293 cells were grown as previously described [32]. The recombinant Toledo_LUC_ strain was kindly provided by Dulal et al. (Department of Microbiology and Molecular Genetics, Rutgers-New Jersey Medical School) [33]. Toledo_LUC_ virus stocks were propagated using MRC-5 cells and titrated as described previously [33].

### 2.3. Radioligand Competition Binding Assay

Stable inducible clones of US28 and CX_3_CR1 cells were seeded at 10,000 cells/well in poly-D-lysine (Invitrogen) coated 96-well tissue culture plates (Nunc). One day after seeding US28 and CX_3_CR1 receptor expression was induced by tetracycline (Invitrogen; 3,6 ng/mL and 5 ng/mL, resp.) aimed at obtaining 5–10% specific binding of the added radioactive ligand. One day after induction, cells were assayed by competition binding for 3 h at 4°C using 20–70 pM ^125^I-CX_3_CL1 as well as unlabeled ligand 10 pM to 100 nM in 50 mM Hepes buffer pH 7.4, supplemented with 1 mM CaCL_2_, 5 mM MgCL_2_, and 0,5% (w/v) bovine serum albumin (BSA) (binding buffer). After incubation, cells were washed twice in ice-cold binding buffer and supplemented with 0,5 M NaCl. Determinations were made in quadruplicate.

### 2.4. Cell Killing Assay

Stable clones of inducible US28 and CX_3_CR1 HEK-293 cells were seeded in poly-D-lysine-coated 48-well tissue culture plates (Nunc) in 300 *µ*L DMEM (Invitrogen). One day after seeding US28 and CX_3_CR1 receptor expression was induced by tetracycline (0,125 *µ*g/mL and 0,25 *µ*g/mL, resp.). One day after induction, a single dose treatment was applied with indicated concentrations of the FTPs (10 pM to 100 nM) and buffer (negative control) and cells were incubated for 24 h at 37°C. To estimate cell health, the cells were incubated with AlamarBlue (Invitrogen) in DMEM without FBS (10% solution) 300 *µ*L per well, for 4 h at 37°C. Data were collected using a Synergy HT plate reader. Determinations were made in quadruplicate.

### 2.5. HCMV Luciferase Assay

MRC-5 (ATCC CCL-171) were seeded in 96-well white tissue culture plates (Nunc) at an initial cell density of 8000 cells/well and infected the next day with Toledo_LUC_ (multiplicity of infection of 0,1 [1 out of 10 cells]). The day after, cells were treated with a single dose of different concentrations of FTPs (10 pM to 1 *µ*M) and buffer (negative control), followed by an incubation for three days at 37°C. On day four after infection, media were exchanged and 100 *µ*L 1x PBS supplemented with MgCl_2_ and CaCl_2_ as well as 100 *µ*L britelite™ plus reagent (Perkin & Elmer) were added. Luciferase activity was measured using a Synergy HT plate reader. Determinations were made in quadruplicate.

### 2.6. Data Analyses

Data analyses were performed using Prism version 6.0.1. Data are expressed as means ± SEM.

## 3. Results

### 3.1. Modification in the Chemokine Domain to Gain More Selectivity for US28

**Figure 2:**
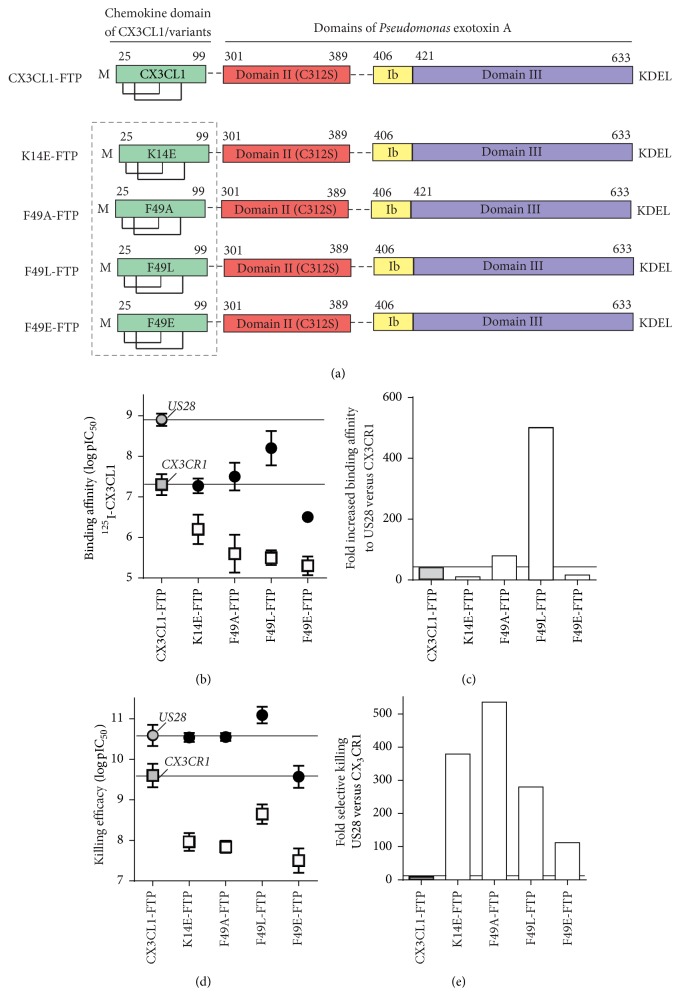
Design, binding, cell killing, and antiviral activity of FTPs with a modified CX_3_CL1 domain. (a) Schematic representation of CX_3_CL1-based FPTs created by fusion of CX_3_CL1 variants to domains of PE. (b) Binding of the prototype CX_3_CL1-FTP (grey symbols) and CX_3_CL1-based FTPs on HEK-293 cells induced to express US28 (black circles) and CX_3_CR1 (white squares). (c) Binding selectivity of CX_3_CL1-FTP and CX_3_CL1-based FTPs determined as fold improved affinity for US28 relative to CX_3_CR1. (d) Cell killing of CX_3_CL1-FTP (grey symbols) and CX_3_CL1-based FTPs on tetracycline induced HEK-293 cells expressing US28 (black circles) and CX_3_CR1 (white squares). (e) Selectivity of CX_3_CL1-FTP and CX_3_CL1-based FTPs determined as fold improved potency in killing US28- relative to CX_3_CR1-expressing cells. Values present IC_50_ values from 3–5 independent biological replicates (b) and (d).

Based on affinity determination of 35 variants of CX_3_CL1 on US28 and CX_3_CR1, we previously identified F49A as the CX_3_CL1 variant with the highest selectivity to US28 versus CX_3_CR1 (affinity selectivity index of 182 determined as IC_50_ for CX_3_CR1 relative to IC_50_ for US28) [7]. Two other CX_3_CL1 variants (K14E and F49L) also turned out to be selective towards US28 with a selectivity index of 39 and 81, respectively, and both maintained high affinity for US28 [7]. Based on these results, the two recombinant CX_3_CL1 variants were fused to PE to create the new fusion-toxin proteins: K14E-FTP and F49L-FTP (Figure 2(a)). As the Ala-substitution of Phe49 resulted in the highest selectivity index, we further explored this position, by the introduction of a glutamic acid, and fused this chemokine with the toxin to create F49E-FTP. The three FTPs were tested for binding to US28 and CX_3_CR1 using ^125^I CX_3_CL1 as radioligand and compared to CX_3_CL1-FTP (the “prototype”) and F49A-FTP (Figure 2(b)). F49L-FTP maintained high affinity for US28, as the affinity to US28 was 5 times increased compared to F49A-FTP (Figure 2(c) and Table 1) [7]. In contrast, F49L-FTPs affinity to CX_3_CR1 was low (in the millimolar range), leading to a selectivity index of 501 (Figure 2(c) and Table 1). Thus, F49L-FTP displayed a ~6,3-fold higher selectivity for binding to US28 relative to CX_3_CR1 compared to F49A-FTP. However, when testing the cell killing activity of the FTPs (Figure 2(d)), F49A-FTP was still more selective in killing US28- versus CX_3_CR1-expressing cells with a 513-fold higher potency on US28- versus CX_3_CR1-expressing cells, whereas F49L-FTP was half as selective with a 275-fold higher potency (Figure 2(e), Table 2). Despite the overall higher binding affinity to US28 as compared to CX_3_CR1 of F49L-FTP, the FTP with the best selectivity profile in killing US28-expressing cells (F49A-FTP) was chosen as lead candidate for further optimization of the nonchemokine parts.

### 3.2. Refinement of the Linker Region with Parts of the Mucin-Like Stalk of CX_3_CL1

**Figure 3:**
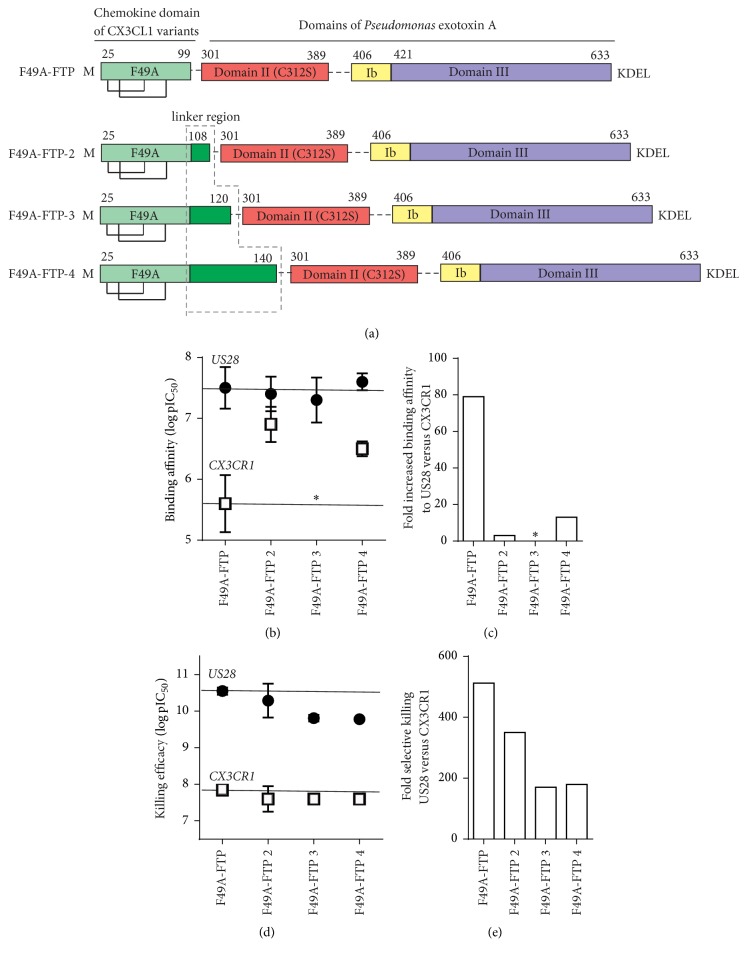
Design, binding, and cell killing of FTPs containing parts of the mucin-like stalk of CX_3_CL1. (a) FTPs with an extended linker consisting of parts of the mucin-like stalk of CX_3_CL1. (b) Binding of F49A-FTP and FTPs from this group on HEK-293 cells induced to express US28 (black circles) and CX_3_CR1 (white squares). The IC_50 _value for F49A-FTP-3 is >10^−6^ M (no binding detectable on CX_3_CR1 expressing cells; marked with a star) and the binding selectivity was therefore not analyzed in (c). (c) Binding selectivity determined as fold improved affinity for US28 relative to CX_3_CR1. (d) Cell killing of F49A-FTP and FTPs from this group on tetracycline induced HEK-293 cells expressing US28 (black circles) and CX_3_CR1 (white squares). (e) Selectivity of F49A-FTP and FTPs from this group determined as fold improved potency in killing US28- relative to CX_3_CR1-expressing cells. Values present IC_50_ values from 3–5 independent biological replicates (b) and (d).

Three FTPs with an extended linker were obtained by adding variable lengths of the mucin-like stalk of CX_3_CL1 (F49A-FTP-2 [9aa], F49A-FTP-3 [21aa], and F49A-FTP-4  [41aa]) (Figure 3(a)). The FTPs maintained high binding affinity to US28 similar to F49A-FTP (Figure 3(b)) but had a reduced selectivity in their binding affinity to US28 versus CX_3_CR1 (Figure 3(c)), as their affinities to CX_3_CR1 increased (Figure 3(b)). Furthermore, the three FTPs had reduced potencies in cell killing of US28-expressing cells proportional to the length of the added mucin-like stalk domain (Figure 3(d)). F49A-FTP-3 and -4 with the longest part of the mucin-like stalk had an up to 3.3-fold lower selectivity in killing US28- versus CX_3_CR1-expressing cells compared to F49A-FTP (Figures 3(d) and 3(e); Table 2). Taken together, these results show that elongation of the linker region with parts of the mucin-like stalk decreases the potency and selectivity of the FTPs compared to F49A-FTP.

### 3.3. Reducing the Linker Region by Removing Domain II of Pseudomonas Exotoxin (PE)

**Figure 4:**
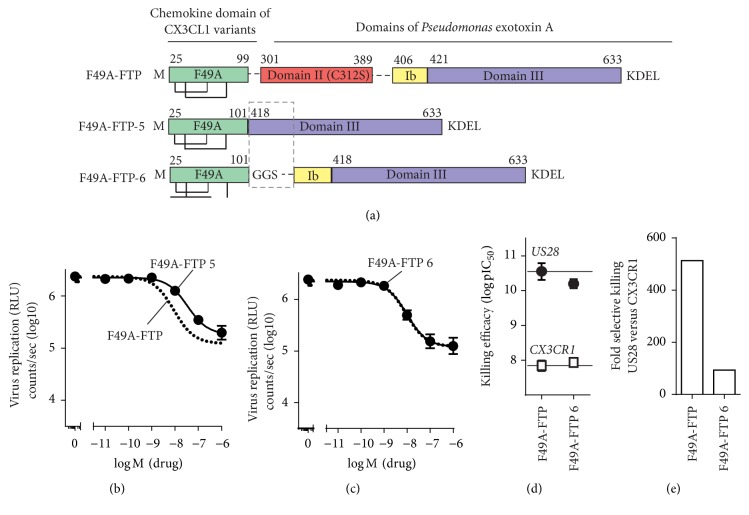
Design, cell killing, and antiviral activity of FTPs without domain II and optional with as GS-linker and Ib domain. (a) F49A-FTP as template for F49A-FTP-5 without domain II between the chemokine domain of CX_3_CL1 and domain III of PE or for F49A-FTP-6 with an additional amino-acid linker (GGS) and part of the Ib domain. (b-c) Inhibition of virus replication measured by luciferase activity of MRC-5 cells infected with Toledo_LUC_ (MOI 0,1) and treated once with F49A-FTP (pos. control; dotted line), F49A-FTP-5, and F49A-FTP-6 (black circles). (d-e) Selectivity of F49A-FTP and F49A-FTP-6 determined as fold improved potency in killing US28- relative to CX_3_CR1-expressing cells. Error bars indicate SEM for 3–5 independent biological replicates.

The furin cleavage site, located in domain II of PE, is important for cleavage of the cytotoxic domains of PE from the chemokine part. We designed two FTPs F49A-FTP-5 and F49A-FTP-6 without domain II and hence without the furin cleavage site. In F49A-FTP-6, we inserted an additional three-amino-acid linker (Gly, Gly, and Ser (GGS)) between the chemokine domain of CX_3_CL1 and the Ib/III domains of PE (Figure 4(a)). The F49A-FTP-5 variant had a reduced antiviral activity, whereas that of F49A-FTP-6 was unchanged compared to F49A-FTP (Figures 4(b) and 4(c); Table 3). As selective killing of US28-expressing cells by the FTP is essential in order to minimize unwanted killing of uninfected host cells expressing CX_3_CR1, we tested F49A-FTP-6-mediated killing of US28- versus CX_3_CR1-expressing cells. Compared to F49A-FTP, F49A-FTP-6 displayed a reduced selectivity for killing US28-expressing cells, as it was ~5.5 times less selective (Figures 4(d) and 4(e) and Table 2). In summary, the domain II of PE is not essential for the antiviral activity, yet its removal decreases the selective killing of the FTPs indicating an altered function of the FTP.

### 3.4. Elongating the Linker with a Full Catalytic Domains of PE

**Figure 5:**
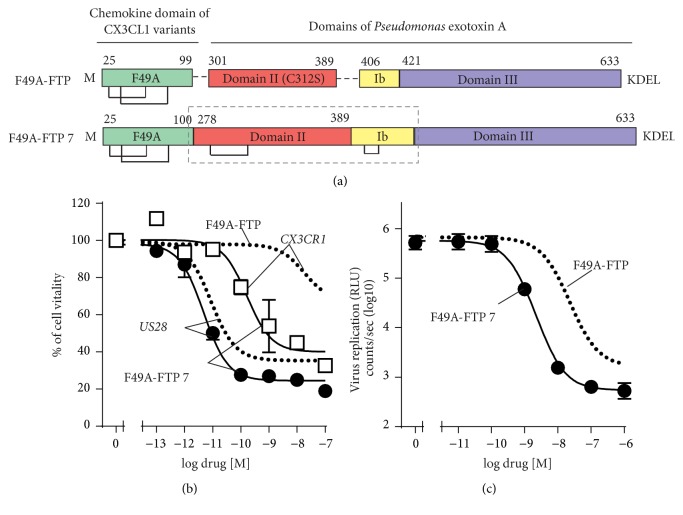
Design, cell killing, and antiviral activity of an FTP with full domains of PE. (a) F49A-FTP-7 with full catalytic active domains of PE. (b) F49A-FTP-7 induced cell killing in comparison to F49A-FTP (dotted line) on tetracycline induced HEK-293 cells expressing US28 (black circles) and CX_3_CR1 (white squares). (c) Inhibition of virus replication measured by luciferase activity of MRC-5 cells infected with Toledo_LUC_ (MOI 0,1) and treated once with F49A-FTP (dotted line) or FTPs from this group (black circles). Error bars indicate SEM for 2–5 independent biological replicates.

As a final step of this study, we investigated if we could increase the antiviral activity of the FTP by fusion of the chemokine with the full catalytic active domains of PE (domains II, Ib, and III) in variant 7, F49A-FTP-7 (Figure 5(a)). This variant lost selectivity by having a much higher potency (40-fold) in killing CX_3_CR1-expressing cells compared to F49A-FTP (Figure 5(b)) and only a slightly higher potency (5,6 fold) in killing US28-expressing cells. The FTP was thereby 7,7 times less selective in killing US28- versus CX_3_CR1-expressing cells with a selectivity index of 66 compared to 513 for F49A-FTP (Table 2). We further determined the antiviral activity of F49A-FTP-7 (Figure 5(c)) and consistent with its improved cell killing, it displayed a higher potency (up to 7,6 times) compared to F49A-FTP. In summary, changes in the enzymatic domains of PE led to a higher antiviral activity of the FTPs but also to more unspecific killing of CX_3_CR1-expressing cells. Based on the results, the prototype FPT “F49A-FTP” with the selective chemokine binding domain and the truncated enzymatic domains (translocation domains II and Ib) turned out to be the best FTP to control virus infections and superior selectivity in killing US28-expressing cells compared to all tested FTPs.

## 4. Discussion

In this study, we generated novel FTPs with the attempt to improve the previously published F49A-FTP [7] in terms of selective killing of US28-expressing cells and antiviral activity. In a systematic approach, we first optimize the chemokine part (that binds to the target receptor). We next varied the linker part (between the chemokine and the toxin) and finally the toxin (variations of PE). F49A-FTP was originally designed based on the selectivity profile (affinity of US28 versus CX_3_CR1) of the chemokine part alone, which, after selection, was fused to the catalytic active domains of PE (Figure 1) [7]. The chemokine system is characterized by redundancy and promiscuity with chemokines that bind more than one receptor and vice versa, but there are also highly selective and monogamous receptor : ligand pairs such as CX_3_CR1 : CX_3_CL1 [34]. For CX_3_CL1 that only binds to one endogenous receptor CX_3_CR1 in addition to the viral US28, it is less complex to employ a rational design strategy to remove binding to the endogenous receptor CX_3_CR1 compared to the US28 binding CC-chemokines that interact with multiple endogenous CC-chemokine receptors [35, 36]. Importantly, the unique mucin-like stalk of CX3CL1 is not necessary for receptor binding as CX3CL1 with this elongation binds similar to CX3CR1 as the soluble chemokine domain of CX3C L1 does [37]. For US28, the affinity of CX3CL1 with its mucin-like stalk is even seven times lower than that of the CX3C chemokine domain alone [28]. However, as US28 still binds the full length CX3CL1 with high affinity (nM) [28], this chemokine is suitable for FTP development as the protein allows for C-terminal modifications and elongations with maintained US28 recognition (Figure 3(b)). Chemokine receptor binding is in general facilitated by interactions between the positively charged chemokine core and the negatively charged extracellular receptor domains, usually including the N-terminus [38, 39]. F49A was picked among 35 CX_3_CL1 variants as the most selective candidate [7]. In the present study, we chose two other selective CX_3_CL1 variants (F49L and K14E), in addition to an extra variant at position 49 (F49E) to create FTPs by fusion of the chemokine fragment with the* Pseudomonas* exotoxin fragments and compared their binding and killing properties to those of F49A-FTP. F49L-FTP had the highest selectivity index in terms of binding, yet due to an improved killing of CX_3_CR1 expressing cells and maintained high killing of US28-expressing cells, the selectivity in terms of killing was reduced. Thus, to create a highly US28-selective FTP as F49A-FTP, it is essential not only to determine its binding affinity to the target receptor but also to investigate its specific killing property. A change of the naturally occurring ligand-receptor complex can influence the molecular properties of the receptor, for example, the rate of receptor internalization or its intracellular trafficking, that is, important receptor features for toxin delivery.

To control HCMV infections, selective targeting of the infected cells is required, but also a high efficacy of the FTP to kill cells before the virus spreads is required. Therefore, we further investigated if we could increase the efficacy of F49A-FTP by modifying the linker region or the catalytic active domains of PE. Elongation of the linker region with parts of the mucin-like stalk of CX_3_CL1 and also deletion of domain II of PE reduced the efficacy of the FTPs in killing US28-expressing cells compared to F49A-FTP. This is consistent with previous studies where eliminating the furin cleavage site by deletion or preventing cleavage with a point mutation in the sites reduced the cytotoxicity of a series of immunotoxins [40].

Besides changes in domain II, changes in the full catalytic domains of PE (domains II, Ib, and III) increased the cytotoxicity and antiviral property of the FTP (F49A-FTP-7). However, the increased killing was observed in both US28- and CX3CR1-expressing cells, indicating a generally improved toxicity of the FTP, again consistent with previous studies [40]. As selective action towards US28 is a perquisite to avoid side effects from inadvertent killing of CX_3_CR1-expressing cells, the prototype F49A-FTP still presents the best candidate therapeutic to treat HCMV infections. To sum up, the presented rational strategy of modifying the chemokine domain, the linker region, and the cytotoxic domains did not improve the prototype FTP. However, as the general understanding of PE toxicity is incomplete, more knowledge is needed. Moreover, questions remain regarding the binding and action of F49A-FTP. So far, the binding of the CX_3_CL1 variants was tested in competition with CX_3_CL1, but it remains to be determined if the modified CX_3_CL1 domain of F49A-FTP can compete against the broad spectrum of CC-chemokines binding to US28, for instance, in an inflammatory situation. Moreover, it remains to be described how the CX_3_CL1-based FTPs act on cells expressing the membrane bound CX_3_CL1, as the soluble form of CX_3_CL1 has been shown to bind to transmembrane CX_3_CL1 with high affinity, that is, with the latter acting as a receptor that thereby influences the communication between cells [41].

Immunotoxins targeting virus-encoded receptors represent promising drugs, not only for HCMV therapy but also for other herpesviruses by targeting their virus-encoded GPCRs. EBV and KSHV infections can cause cancer and targeting of their GPCRs could be a novel anticancer treatment strategy. The broad-spectrum chemokine receptor ORF74 from KSHV thus seems highly suitable for immunotoxin targeting, as this receptor (1) can induce the onset of Kaposi's-like lesions [42, 43], (2) has a defined chemokine ligand profile [44, 45], and (3) is internalized in response to human CXCL-1 and -8 [46]. As such, using immunotoxins designed to target KSHV-infected cells could be a valid approach to efficiently kill KSHV-infected cells. With respect to the EBV-encoded BILF1 receptor, its pronounced cell surface expression [13, 47], constitutive internalization [17], and expression during the lytic virus replication cycle, but also in latency [48], indicate that this receptor is a promising drug target suitable for immunotoxin targeting delivery. A drawback is that EBV-BILF1 is an orphan receptor (i.e., with no known ligands), but new technologies including nanobody and monobody design could offer highly specific ligands for immunotoxin targeting of EBV-BILF1. In addition to EBV-BILF1, immunotoxin targeting of the endogenous receptor EBI2 [49, 50] that is upregulated upon infection with EBV could be a future strategy for anti-EBV treatment. The role of EBI2 in the EBV life cycle is still uncertain [51], but if EBV benefits from high EBI2 expression, then EBI2 could represent another drug target to control EBV-associated diseases. However, as EBI2 is not a viral protein, more side effects would be a risk factor as the receptor is expressed on a variety of immune cells (B-cells, T-cells, macrophages, dendritic cells, and many others) [52–54].

## 5. Conclusion

Immunotoxin based antiviral drugs offer a novel antiviral mechanism for combination therapy and for treating infections that have become resistant to the current first-line intervention. Here, we show that a CX_3_CL1-based FTP targeting the HCMV-encoded GPCR US28 can be modified for highly effective killing of virus-infected cells by modifying the three structural domains of the FTP (the chemokine domain of CX_3_CL1, the linker region, and the catalytic active domains of PE). By inserting single point mutations in the core domain of CX_3_CL1, the FTP loses affinity for CX_3_CR1, but not for the virus-encoded receptor US28. Changes in the linker region do not improve the activity of FTP, whereas changes in the catalytic active domains of PE increase the killing efficacy for US28-expressing cells and thereby the antiviral activity. Thus, CX_3_CL1-based FTP can be used as scaffold to create highly efficient and selective FTPs to control HCMV infections. As several other virus-exploited GPCRs have been described, the principle of antiviral therapy targeting these receptors may not be limited to US28 for the targeting of HCMV but may be expanded to the targeting of ORF74 for KSHV treatment and BILF1 and/or EBI2 for the treatment of EBV-mediated diseases.

## Figures and Tables

**Table 1:** Homologous binding experiments testing the binding affinity of the FTPs.

	*US28*/^125^I-CX_3_CL1^*∗∗*^		*CX*_*3*_ *CR1*/^125^I-CX_3_CL1^*∗∗*^		Binding selectivity
	log IC_50_ ± SD	IC_50_ [nM]	log IC_50_ ± SD	IC_50_ [nM]	CX_3_CR1 versus US28 (CX_3_CL1)
CX_3_CL1-FTP	−8.9 ± 0.26	1.3	−7.3 ± 0.45	50	**40**
K14E-FTP	−7.2 ± 0.31	63	*−6.2* ± *0.63*	*631* ^*∗*^	***10***
F49A-FTP	−7.5 ± 0.59	32	*−5.6* ± *0.81*	*2512* ^*∗*^	***79***
F49L-FTP	−8.2 ± 0.95	6.3	*−5.5* ± *0.36*	*3162* ^*∗*^	***501***
F49E-FTP	−6.5 ± 0.07	316	*−5.3* ± *0.40*	*5012* ^*∗*^	***16***
F49A-FTP 2	−7.4 ± 0.49	40	*−6.9* ± *0.50*	*126* ^*∗*^	***3***
F49A-FTP 3	−7.3 ± 0.64	50	*>5,0*		*—*
F49A-FTP 4	−7.6 ± 0.24	25	*−6.5* ± *0.21*	*316* ^*∗*^	***13***
F49A-FTP 7	−7.3 ± 0.38	50	*−6.6* ± *0.20*	*251* ^*∗*^	***5***

^*∗*^IC_50_ values of the FTPs bound to CX_3_CR1 have been estimated from a partial curve.

^*∗∗*^IC50 value estimated from 3-4 experiments.

**Table 2:** Cell killing activity of the FTPs.

	*US28 exp. cells* ^*∗∗*^		*CX*_*3*_ *CR1 exp. cells* ^*∗∗*^		Selectivity
	log IC_50_ ± SD	IC_50_ [nM]	log IC_50_ ± SD	IC_50_ [nM]	US28 versus CX_3_CR1
CX_3_CL1-FTP	−10.6 ± 0.52	0.026	*−*9.6 ± 0.45	0.25	**10**
K14E-FTP	−10.5 ± 0.25	0.029	*−*8.0 ± 0.22	11	**380**
F49A-FTP	−10.6 ± 0.24	0.028	*−*7.8 ± 0.15	14	**513**
F49L-FTP	−11.1 ± 0.41	0.008	*−*8.7 ± 0.24	2.2	**275**
F49E-FTP	−9.6 ± 0.47	0.27	*−7.5* ± *0.30*	*32* ^*∗*^	***117***
F49A-FTP 2	−10.3 ± 0.08	0.051	*−7.8* ± *0.30*	*16* ^*∗*^	***309***
F49A-FTP 3	−9.8 ± 0.16	0.15	*−7.6* ± *0.20*	*25* ^*∗*^	***162***
F49A-FTP 4	−9.8 ± 0.06	0.17	*−7.6* ± *0.30*	*25* ^*∗*^	***151***
F49A-FTP 5	n.a.		n.a.		—
F49A-FTP 6	−9.5 ± 0.20	0.34	*−7.5* ± *0.10*	*32* ^*∗*^	***93***
F49A-FTP 7	−11.3 ± 0.24	0.005	*−*9.5 ± 0.49	0.35	**66**

^*∗*^log IC50 values are estimated from a partial curve.

^*∗∗*^IC50 value estimated from 3–6 experiments.

**Table 3:** Antiviral activity of the FTPs.

	Potency^*∗*^	
	log IC_50_ ± SEM	IC_50_ [nM]
CX_3_CL1-FTP	−9.3 ± 0.36	0.52
K14E-FTP	−8.1 ± 0.09	8.9
F49A-FTP	−7.7 ± 0.05	20
F49A-FTP 2	n.a.	n.a.
F49A-FTP 3	n.a.	n.a.
F49A-FTP 4	n.a.	n.a.
F49A-FTP 5	−7.5 ± 0.24	30
F49A-FTP 6	−8.0 ± 0.10	9.3
F49A-FTP 7^*∗∗*^	−8.6 ± 0.13	2.6

^*∗*^IC50 value estimated from 3–5 experiments.

^*∗∗*^IC50 value estimated from 2 experiments.
